# Structural instability impairs function of the UDP‐xylose synthase 1 Ile181Asn variant associated with short‐stature genetic syndrome in humans

**DOI:** 10.1002/1873-3468.70277

**Published:** 2026-01-13

**Authors:** Tuo Li, Pedro A. Sánchez‐Murcia, Bernd Nidetzky

**Affiliations:** ^1^ Institute of Biotechnology and Biochemical Engineering Graz University of Technology Austria; ^2^ Division of Medicinal Chemistry, Otto‐Loewi Research Center Medical University of Graz Austria; ^3^ BioTechMed‐Graz Austria; ^4^ Austrian Centre of Industrial Biotechnology (acib) Graz Austria

**Keywords:** linkeropathy, protein dimer, proteoglycan, stability, tetrasaccharide linker, UDP‐xylose, UDP‐xylose synthase UXS1

## Abstract

Glycosaminoglycan assembly on proteoglycans involves a common tetrasaccharide linker that starts with xylose attached to a serine on the protein. Defective linker biosynthesis caused by a missense mutation of human UDP‐xylose synthase (hUXS1) is associated with connective tissue disorders characterized by skeletal abnormality and short stature. The Ile181Asn variant of hUXS1 was reported as inactive in releasing UDP‐xylose from UDP‐glucuronic acid. Here, we show that Ile181Asn‐hUXS1 exhibited catalytic properties similar to the wild‐type enzyme but featured a significant decrease in stability, expressed in melting temperature lowered from 48.2 °C to 35.2 °C. At 37 °C, Ile181Asn‐hUXS1 was ~10‐fold less stable and more prone to precipitation than wild‐type hUXS1. The loss of function in Ile181Asn‐hUXS1 is thus explained by instability, consistent with molecular dynamics simulations predicting structural destabilization.

**Impact statement**

The Ile181Asn variant of human UDP‐xylose synthase (hUXS1), associated with a short‐stature genetic syndrome, has previously been reported as inactive. We show here with experiments and molecular simulations that hUXS1 malfunction arises from structural instability rather than from a catalytic defect.

## Graphical abstract

The Ile181Asn variant of human UDP‐xylose synthase (hUXS1), associated with a short‐stature genetic syndrome, has previously been reported as inactive. Our findings demonstrate that Ile181Asn‐hUXS1 retains catalytic activity similar to the wild‐type but exhibits reduced stability, a looser oligomeric state, and an increased tendency to precipitate. Thus, its malfunction arises from structural instability rather than catalytic deficiency, consistent with molecular dynamics simulations.

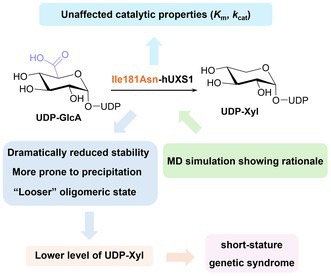

## Abbreviations

**DSF**, differential scanning fluorimetry

**ECM**, extracellular matrix

**GAG**, glycosaminoglycan

**SDR**, short‐chain dehydrogenases/reductases

**UDP‐GlcA**, UDP‐glucuronic acid

**UDP‐Xyl**, UDP‐xylose

**UXS**, UDP‐xylose synthase (EC4.1.1.35)

**Fig. 1:**
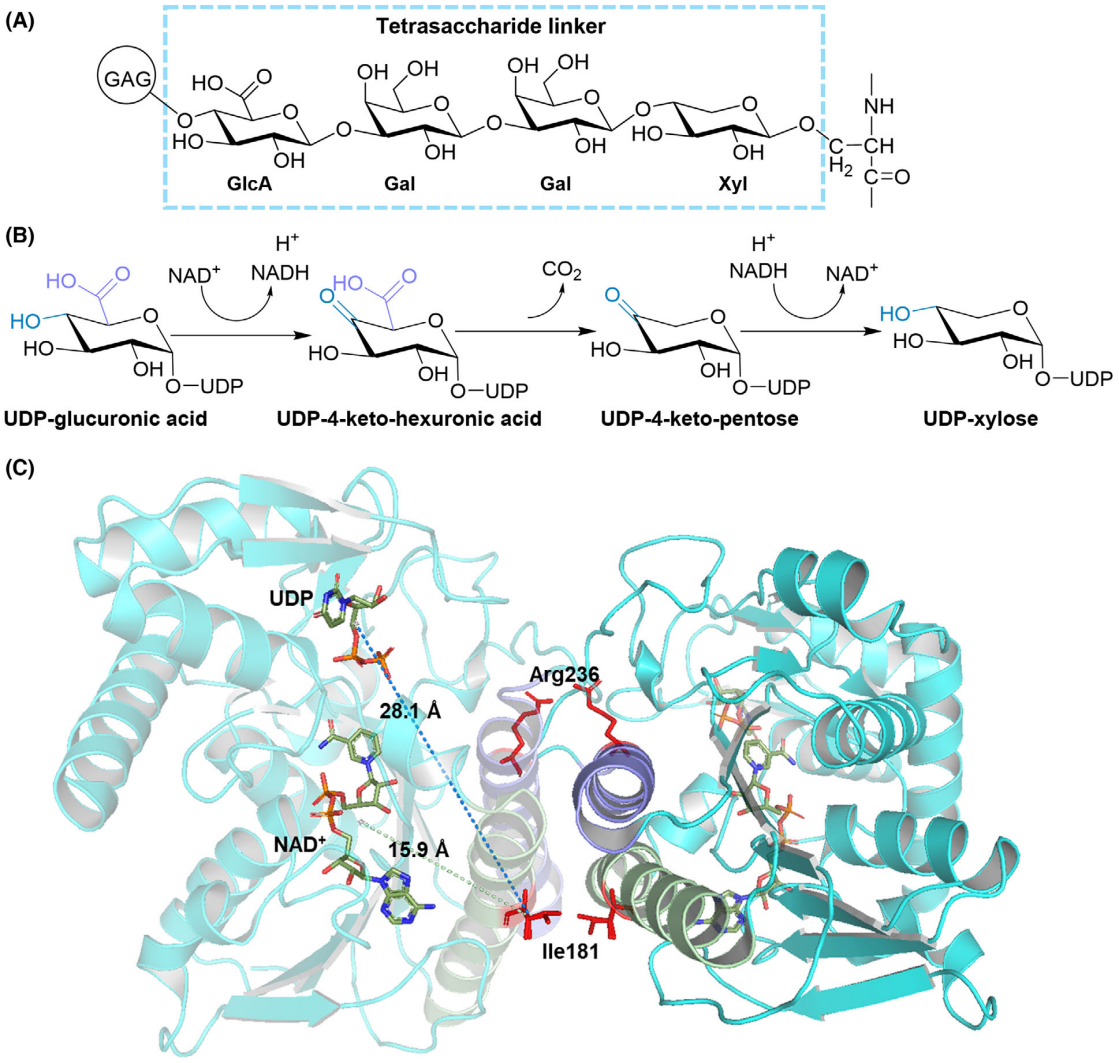
The role of UXS (UDP‐xylose synthase) in proteoglycan biosynthesis. (A) Structural organization of a proteoglycan, featuring GAG (glycosaminoglycan) chain attached via a common tetrasaccharide linker to a serine of the protein backbone. (B) Proposed catalytic reaction of UXS. (C) Three‐dimensional structure of the hUXS1 (human UXS 1) dimer in complex with NAD^+^ and UDP (PDB ID: 2B69), highlighting the position of Ile181 and Arg236 whose individual substitutions give rise to clinical phenotypes in humans.

Proteoglycans are key components of the extracellular matrix (ECM) in connective tissues [1, 2]. They consist of a core protein covalently tethered with long glycosaminoglycan (GAG) chains [3, 4]. A common tetrasaccharide linker, starting with a xylose residue attached to a serine on the protein backbone, connects the GAG chains to the protein (Fig. 1A) [5, 6]. UDP‐xylose (UDP‐Xyl) is the donor of the xylose building block in linker biosynthesis [7, 8]. It is derived from UDP‐glucuronic acid (UDP‐GlcA) through the reaction of UDP‐xylose synthase (UXS; other name: UDP‐glucuronic acid decarboxylase/carboxy‐lyase; EC4.1.1.35) [7, 9], as shown in Fig. 1B. UXS is widely distributed among species and represents one of the most highly conserved proteins in nature [10, 11]. UXS belongs to the short‐chain dehydrogenase/reductase (SDR) protein superfamily and defines a distinct family of the so‐called extended SDRs that perform multistep redox‐based transformations on sugar nucleotide substrates [12, 13].

The term ‘linkeropathy’ refers collectively to a heterogeneous group of genetic syndromes, caused by mutations in the biosynthetic genes for the tetrasaccharide linker and characterized by a spectrum of skeletal and connective tissue disorders [14, 15]. Clinical features include, or combine, short stature, congenital contractures, dislocations, dysplasia with joint laxity, and more [16, 17]. Recently, a severe phenotype of short‐limbed short stature and skeletal dysplasia was linked to an Ile186Asn variant of the human UXS (hUXS1) [18]. Previous studies have identified several hUXS1 transcript variants of hUXS1 [19]. The Ile186Asn mutation was found in the splice variant (425 aa) of the well‐characterized native transcript (420 aa) [7, 20, 21], which contains five additional amino acids (SFLLN, residues 41–45) (Fig. S1) in the membrane domain. A truncated version of native transcript (residues 85–402) containing the main catalytic fragment has been characterized *in vitro* to validate the function of the Ile186Asn variant (referred to as Ile181Asn in this study) [18]. The purified Ile181Asn‐hUXS1 was shown to have lost all biological activity for converting UDP‐GlcA into UDP‐Xyl [18]. The hUXS1 structure shows Ile181 located far away from the active site (> 15 Å) at the interface of the dimeric enzyme (Fig. 1C). An active hUXS1 tetramer has also been reported [22], but this is essentially a dimer of dimers. The substitution of isoleucine with asparagine was presumed to disrupt the intermolecular forces between the monomers, thereby leading to malfunction [18]. Previously, another so‐called *man o’ war* (*mow*) mutation was found in hUXS1 (Arg236His) located at the dimer interface (Fig. 1C) [23]. Arg236His‐hUXS1 was shown to involve greater conformational flexibility than wild‐type hUXS1, with the result that various oligomer states (dimer, tetramer, hexamer) become populated by the variant enzyme in solution [23]. The mutation generates a specific defect in UXS catalysis, that is, the inability to promote the reduction step of the overall enzymatic reaction [23]. This defect results in an abortive catalytic cycle of Arg236His‐hUXS1 in which UDP‐4‐keto‐pentose and NADH are released as products when exogenous NAD^+^ is provided [23]. Collectively, these studies [10, 18, 23] show that residue substitutions in the dimer interface region can affect hUXS1 function.

This study was performed to characterize the biochemical properties of Ile181Asn‐hUXS1 in greater detail than previously reported [18]. The evidence for the *mow* variant Arg236His‐hUXS1 [23] supports the notion that deeper characterization of Ile181Asn‐hUXS1 could provide important insight. We show with experimental characterization and molecular dynamics (MD) simulations that Ile181Asn‐hUXS1 is structurally less stable than wild‐type hUXS1. Both enzymes are identical in their catalytic behavior (i.e., steady‐state kinetic parameters, product formation), yet Ile181Asn‐hUXS1 is more prone to denaturation and inactivation than wild‐type enzyme. The melting temperature *T*_m_ of 35.2 °C for Ile181Asn‐hUXS1 is lowered by 13 °C compared to the *T*_m_ of wild‐type hUXS1. At physiological temperature of 37 °C, the observed dysfunction of Ile181Asn‐hUXS1 is thus explainable by low protein stability.

## Materials and methods

### Materials

UDP‐GlcA (> 98%), NAD^+^ (> 99%), and SYPRO® Orange (5000× stock solution, dye) were from Sigma Aldrich (Vienna, Austria). PrimeSTAR® Max DNA Polymerase (2× Premix) was from Takara Bio (London, United Kingdom). Protein molecular mass standards were from GE Healthcare UK Limited (Chalfont Saint Giles, United Kingdom). The UDP‐Xyl (> 99%) and UDP‐4‐keto‐pentose (> 99%) were obtained by enzymatic synthesis done in a previous study [24]. All other chemicals were of the highest available purity from Sigma Aldrich, Roth (Karlsruhe, Germany), or Merck (Vienna, Austria).

### Enzyme production

Based on previous research [7], this study utilized an optimized truncated form of hUXS1 (NM_025076.5, residues 85–402) featuring an N‐terminal 6 × His tag and a tobacco etch virus (TEV) protease cleavage site (Fig. S2). The site‐directed substitution of Ile181 by Asn was introduced by a modified QuikChange protocol [24], using PrimeSTAR® Max DNA Polymerase for DNA amplification. The primers were designed as follows: forward primer (5′‐3′), CTGAAAACTAACACCAACGGCACCTTGAACATG, and reverse primer (5′‐3′), CATGTTCAAGGTGCCGTTGGTGTTAGTTTTCAG. Both hUXS1 wild‐type and Ile181Asn variant were produced by expression in *E. coli* BL21 (DE3) cells that harbored the pET‐derived expression vector p11a. After expression in Lysogeny Broth medium, His‐tagged target proteins were purified using a HisTrap™ HP column (5 mL resin, Cytiva, Uppsala, Sweden) following the previously described method [18]. The size and purity of these enzymes were confirmed by SDS‐PAGE (Fig. S3).

### Determination of kinetic parameters

Reactions were carried out at varying concentrations of UDP‐GlcA (1.0–50 mm) and fixed concentrations of NAD^+^ (0.50 mm) and enzyme (0.20 mg·mL^−1^; 5.2 μm) in a total volume of 100 μL at pH 8.0 and 25 °C (or 37 °C). After incubation for an appropriate duration (up to 10 min or 40 min), the reactions were quenched with methanol (80% (v/v) final concentration) and centrifuged at 21 130 ***g*** for 20 min. The resulting supernatants were analyzed by HPLC. All experiments were performed in duplicate. Reaction rates were calculated based on the formation of UDP‐Xyl over time, using the linear portion of the corresponding time courses (Figs S4 and S5). The values of *K*_m_ and *k*_cat_ were determined by fitting the data with a hyperbolic Michaelis–Menten model, using nonlinear least‐squares analysis in Origin 2021. The *k*_cat_ was calculated from the maximum reaction rate (*V*_max_) and the molar enzyme concentration [E], using the equation *k*_cat_ = *V*_max_/[E]. The value of [E] was derived from the mass‐based protein concentration and the molecular weight of the hUXS1 polypeptide (*M*_r_ = 38 442).

### HPLC analysis

The separation of UDP‐GlcA and UDP‐Xyl, as well as the analysis of NAD^+^, were performed using a Shimadzu Prominence HPLC‐UV system (Shimadzu, Korneuburg, Austria) equipped with a Kinetex C18 column (5 μm, 100 Å, 50 × 4.6 mm). Analyses were conducted under isocratic conditions at a flow rate of 2.0 mL·min^−1^ for 5 min (UDP‐GlcA, UDP‐Xyl) or 2 min (NAD^+^). The mobile phase consisted of 5% acetonitrile (for UDP‐GlcA and UDP‐Xyl) or 5% methanol (for NAD^+^) and 95% tetrabutylammonium bromide (TBAB) buffer (40 mm TBAB, 20 mm potassium phosphate, pH 5.9). Separation of UDP‐Xyl and UDP‐4‐keto‐pentose (Fig. S6) was performed using a Kinetex C18 column (5 μm, 100 Å, 250 × 4.6 mm) under isocratic conditions with a flow rate of 0.80 mL·min^−1^ over 60 min, employing a mobile phase composed of 10% methanol and 90% TBAB buffer. UV detection was at 262 nm. The consumption of the substrate (UDP‐GlcA) was assumed to be equivalent to the formation of the single product (UDP‐Xyl), under the fact that no side reactions or hydrolysis occurred. The relative ratio of the reaction product was determined by calculating the proportion of the UDP‐Xyl peak area to the total peak area of UDP‐Xyl and UDP‐GlcA in the HPLC chromatograms. The concentration of the product (UDP‐Xyl) was subsequently estimated by multiplying the initial concentration of UDP‐GlcA by the corresponding relative ratio.

### Differential scanning fluorimetry (DSF)

hUXS1 wild‐type and Ile181Asn variant were diluted to a final concentration of 5.0 μm in potassium phosphate buffer (50 mm, pH 8.0). Triplicate 45 μL samples of the wild‐type protein, the Ile181Asn variant, or buffer alone (negative control) were each mixed with 5.0 μL of SYPRO® Orange dye (200×). The mixtures were carefully transferred to a 96‐well PCR plate (Bio‐Rad, Hercules, CA, USA), ensuring the absence of air bubbles. Thermal denaturation was performed using a Bio‐Rad CFX Connect Real‐Time PCR Detection System, applying a temperature gradient from 20.0 °C to 95.0 °C in 0.5 °C increments, with each temperature step maintained for 30 s. DSF data were analyzed using Bio‐Rad CFX Maestro software.

### Thermal inactivation

The hUXS1 wild‐type and Ile181Asn variant were diluted to either 1.0 mg·mL^−1^ or 0.10 mg·mL^−1^ using 50 mm potassium phosphate buffer (pH 8.0) and incubated at 37 °C in a total volume of 100 μL, with all conditions consistent except for protein concentration. At designated time points, samples from the incubated solutions (40 μL for 1.0 mg·mL^−1^ and 70 μL for 0.10 mg·mL^−1^) were withdrawn and immediately placed on ice, followed by the assessment of residual enzymatic activity. Activity assays (100 μL) were conducted at 25 °C without agitation, using a reaction mixture containing 5.0 mm UDP‐GlcA, 0.50 mm NAD^+^, and pre‐incubated enzyme solution, in potassium phosphate buffer (50 mm, pH 8.0). At the desired time points, reaction mixtures were quenched and analyzed by HPLC as described in the section ‘Determination of kinetic parameters’. Initial UDP‐Xyl formation rates were determined from the linear portions of the respective time courses and used to calculate relative activity, with the initial rate of hUXS1 wild‐type at 0 min defined as 100%. All measurements were conducted in duplicate. The associated rate constants (*k*_in_) were determined as the absolute values of the slopes derived from linear regression analyses of ln(relative activity) plotted against time (Fig. S7).

### Light scattering measurements

Light scattering measurements were performed using a fluorescence spectrometer (F‐4500, Hitachi, Tokyo, Japan) to assess the precipitation behavior of hUXS1 wild‐type and Ile181Asn variant incubated at 37 °C. Samples (1.0 mL) were prepared at final concentrations of 1.0 mg·mL^−1^ or 0.10 mg·mL^−1^ in 50 mm potassium phosphate buffer (pH 8.0) and incubated in 2 mL Eppendorf tubes using a ThermoMixer (Eppendorf, Hamburg, Germany) without agitation. At designated time points, samples (undiluted for 0.10 mg·mL^−1^ and diluted 50× for 1.0 mg·mL^−1^) were transferred to a 1 cm path length quartz cuvette, mixed thoroughly, and analyzed by excitation wavelength scans (Fig. S8). Excitation spectra were recorded from 600 to 660 nm at a scanning speed of 240 nm·min^−1^, with the emission wavelength fixed at 635 nm, and the excitation slit width set to 5.0 nm. All measurements were performed in triplicate.

### Size exclusion chromatography (SEC)

SEC was performed with an ÄKTA Go system (Cytiva) connected to a HiLoad 16/6 Superdex 200 prep grade column (GE Healthcare). Native protein samples (12 mg wild‐type or 8 mg Ile181Asn variant) were eluted with 10 mm HEPES buffer (pH 7.45) containing 150 mm NaCl and 0.10 mm tris(2‐carboxyethyl) phosphine (TCEP), at a flowrate of 1.0 mL·min^−1^ and maintained at ~20 °C. The apparent molecular mass was calculated from a calibration curve (Fig. S9) prepared with protein standards (6500–75 000 Da). The main peak and shoulder peak fractions of the Ile181Asn‐hUXS1 sample, corresponding to elution volumes of 70–80 mL and 80–90 mL, respectively, were collected and concentrated using Vivaspin ultrafiltration devices (10 000 MWCO PES; Sartorius, Goettingen, Germany), for activity assays (Fig. S10). To examine the effect of protein concentration on the oligomerization state, varying amounts of protein (0.8–4.5 mg) were loaded for SEC analysis. To assess the oligomerization state after incubation, hUXS1 wild‐type (12 × 1.0 mL, 1.0 mg·mL^−1^) and Ile181Asn‐hUXS1 (18 × 1.0 mL, 1.0 mg·mL^−1^) samples were incubated at 37 °C for 2 h and 30 min, respectively. Following centrifugation (21 130 ***g***, 4 °C, 20 min) to remove insoluble material, the concentration of soluble protein in the supernatant was determined by measuring absorbance at 280 nm using a Nanophotometer N50 (IMPLEN, Munich, Germany), applying a calculated molar extinction coefficient of 37 485 m ^−1^·cm^−1^ based on the amino acid sequence. After concentration to approximately 1.0 mL using Vivaspin ultrafiltration tubes (10 kDa MWCO, as described above), the treated samples were subjected to SEC analysis.

### MS analysis (native mass spectrometry analysis)

Protein samples (50 pmol, 5 μL at 10 μm concentration) were desalted on an AdvanceBio SEC 300 Å (2.7 μm, 4.6 × 50 mm; Agilent, Vienna, Austria) size‐exclusion HPLC column equilibrated in 150 mm ammonium acetate (pH 7.0). A Shimadzu Nexera UHPLC system was used for automated injections and desalting via the column at a flow rate of 0.2 mL·min^−1^. The eluting proteins were directly injected into a Bruker maxis II ultra‐high resolution Q‐TOF device (Bruker Daltonics Inc., Billerica, MA, USA), and the elution volumes containing salts were diverted to the waste valve. Mass spectra were acquired in an m/z range between 500 and 10 000 with 2.5 bar nebulizer pressure and 8 L·min^−1^ dry gas setting. The source temperature was set to 325 °C and is CID energy to 100 eV. Transfer time and pre pulse storage were optimized for nMS with 180 μs and 40 μs, respectively. Calibration of the mass spectrometer in the m/z range from 1822 to 5460 was performed using the ESI‐tune mix at elevated concentrations allowing assignments of dimeric and trimeric species. Raw data (Fig. S11) were analyzed using DataAnalysis (Bruker) and maximum entropy deconvolution of the protein signals.

### Enzyme‐bound NAD ^+^

For studying the concentration of NAD^+^ in the hUXS1 wild‐type and Ile181Asn variant, the enzyme (10 μL, 30 mg·mL^−1^) was denatured by incubating with 40 μL of methanol for 2 h at room temperature. After adding 200 μL of water, the precipitated enzyme was removed by centrifugation (21 130 ***g***, 30 min, 4 °C), and the supernatant was analyzed on HPLC. The amount of released NAD^+^ was calculated based on a calibration curve (Fig. S12), where defined concentrations of NAD^+^ were prepared in water and directly used for measurements.

### MD simulations

The Cartesian coordinates of hUXS1 were obtained from the crystallographic structure of the enzyme in complex with NAD^+^ and UDP (PDB ID: 2B69) [7]. All atoms of the system were parameterized using AMBER atom types. For UDP, RESP charges were derived from the electrostatic potential calculated on an optimized geometry (HF/6‐31G*//BP86‐D3/def2‐SVP + SCRF(water)) using Gaussian16, rev. C1 [25]. Force field parameters for NAD^+^, calculated by U. Ryde, were obtained from the Bryce AMBER Database [26]. For the glycosyl moieties of the system, the GLYCAM_06j‐1 parameters were used [27]. The dimeric hUXS1 was embedded in a box of OPC water molecules [28], and the system was neutralized with 20 Na^+^ ions. Energy minimization was performed in three consecutive steps, allowing relaxation of hydrogen atoms, all molecules except the solute, and the entire system, respectively. The minimized system was then heated from 100 to 300 K at constant volume (NVT ensemble) over 200 ps using a Langevin thermostat (friction coefficient of 1 ps^−1^) at constant pressure. All solute atoms were constrained using a harmonic force constant of 200 kcal·mol^−1^·Å^−2^. These constraints were gradually released in six steps (40, 30, 20, and 10 kcal·mol^−1^·Å^−2^ under the NVT ensemble, and 10 and 0 kcal·mol^−1^·Å^−2^ under constant pressure, NPT). The equilibrated system was further simulated for 2.5 μs (five independent simulations of 0.5 μs each) with a time step of 2 fs. Periodic boundary conditions were applied. SHAKE was used to constrain all bonds involving hydrogen atoms, and long‐range electrostatic interactions were computed using the Particle Mesh Ewald (PME) method [29]. All simulations were performed with AMBER24 with *pmemd*.cuda on GPU. The MD trajectories were analyzed with *cpptraj*. To calculate the change in free energy upon mutation (∆∆G), the *PositionScan* function of FoldX 2025 was used [30]. Five hundred snapshots from the MD simulations were analyzed, excluding the first 100 ns of each trajectory. Bond‐to‐bond propensity scores were computed from the crystallographic structure of the enzyme using the ProteinLens server [31]. The likelihood score was calculated as a mean value of the five GPU‐accelerated protein language models for zero‐shot inference of mutational effects on protein function ESM‐1v [32].

## Results and discussion

### Biochemical characterization of Ile181Asn‐hUXS1

Wild‐type and Ile181Asn forms of hUXS1 were obtained from *Escherichia coli* overexpression cultures in isolated protein amounts (per L culture) of 23 mg and 13 mg, respectively. Both enzymes were truncated to remove residues 1–84 (at the N‐terminus) that comprise the membrane anchor of the full‐length native hUXS1, and residues 402–420 for a predicted random coil at the C‐terminus. They were equipped with an N‐terminal His‐tag used in purification according to a previous report [7]. The isolated enzymes were pure by the criterion of a single protein band in SDS‐PAGE (Fig. S3) and showed the mass (~38 kDa) expected for the truncated hUXS1 (residues 85–402; calculated mass: 38442 Da).

**Fig. 2:**
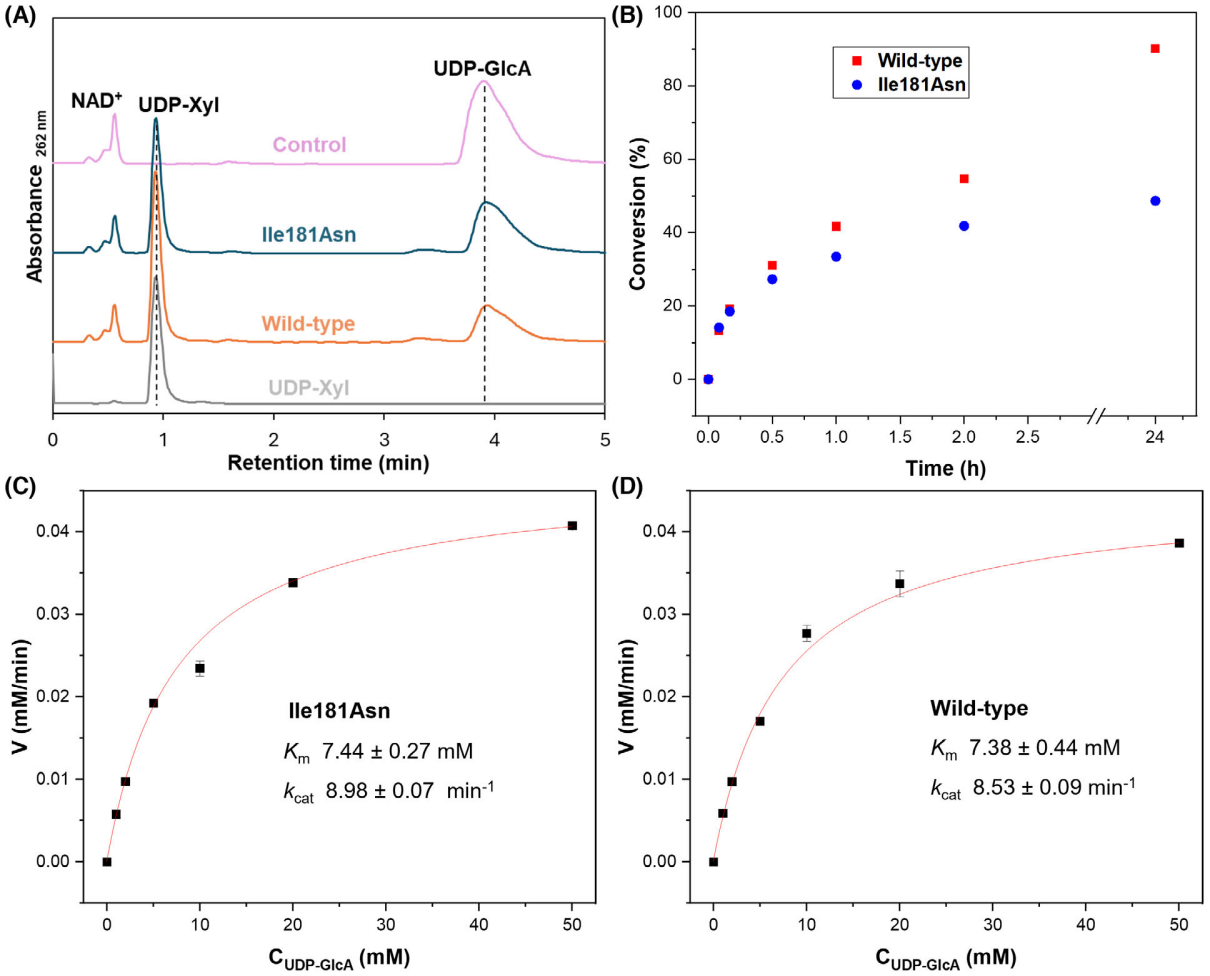
Biochemical characterization of Ile181Asn‐hUXS1. (A) HPLC absorbance traces showing conversion of UDP‐GlcA (UDP‐glucuronic acid) into UDP‐Xyl (UDP‐xylose). (B) Time course analysis showing conversion based on UDP‐Xyl formation. Reactions (100 μL) contained 5.0 mm UDP‐GlcA, 0.50 mm NAD^+^, and 1.0 mg·mL^−1^ enzyme in potassium phosphate buffer (50 mm, pH 8.0) and were carried out at 37 °C without agitation. (C, D) Michaelis–Menten plots for the determination of kinetic parameters for Ile181Asn‐hUXS1 (C) and wild‐type hUXS1 (D). Assays contained 0.20 mg·mL^−1^ enzymes, 1.0–50 mm UDP‐GlcA, 0.50 mm NAD^+^, and were performed at 25 °C. The initial reaction rates were calculated from the linear portion (up to 40 min) of the corresponding time courses (Fig. S4). Symbols show the data and the line is a hyperbolic fit. hUXS1, human UDP‐xylose synthase 1.

Purified enzymes (1.0 mg·mL^−1^; 26 μm) were incubated with UDP‐GlcA (5.0 mm) and NAD^+^ (0.50 mm) at 37 °C in 50 mm potassium phosphate buffer (pH 8.0). Samples were taken at certain times and analyzed by HPLC referenced against authentic standards of UDP‐GlcA, UDP‐Xyl, and UDP‐4‐keto‐pentose. Reaction of wild‐type UXS1 gave UDP‐Xyl release exactly proportional to UDP‐GlcA consumption, while Ile181Asn‐hUXS1 was also active in converting UDP‐GlcA into UDP‐Xyl (Fig. 2A). The UDP‐4‐keto‐pentose intermediate, which was clearly separated from UDP‐Xyl by the HPLC methods used, was not detected (Fig. S6). The amount of UDP‐Xyl released was matched exactly to the UDP‐GlcA consumed. The initial UDP‐Xyl formation rates were similar for Ile181Asn‐hUXS1 and wild‐type enzyme (Fig. 2B), implying that both enzymes exhibit comparable specific activities, determined as ~0.14 U·mg^−1^. These results contrast sharply with the previous report on Ile181Asn‐hUXS1 [18], showing that no UDP‐Xyl was produced from UDP‐GlcA by the variant.

We then characterized Ile181Asn‐hUXS1 in steady‐state kinetic studies of UDP‐GlcA conversion. Initial rates (≤ 15% substrate conversion) were acquired at varied concentrations of UDP‐GlcA at 25 °C or 37 °C. Results in Fig. 2C; Fig. S5c and their comparison with the kinetic data for wild‐type hUXS1 (Fig. 2D; Fig. S5d) suggest that the catalytic properties of Ile181Asn‐hUXS1 were the same as those of the wild‐type enzyme. The values of the catalytic coefficient (*k*_cat_) and the Michaelis constant (*K*_m_) were identical for both enzymes within the limits of the experimental error. The results obtained here for the wild‐type hUXS1 were in agreement with earlier work [7]. Low enzymatic activity (~0.8 mU·mg^−1^, Fig. S13) was observed for both the wild‐type and the Ile181Asn variant under the assay conditions described in the previous study on Ile181Asn‐hUXS1 [18]. This low activity is due to the high *K*_m_ value (~8 mm) of the enzymes and the relatively low UDP‐GlcA concentration (0.02 mm) used in the assay. Enzyme specificity for product formation was unaltered by the change in UDP‐GlcA substrate concentration in the range used for kinetic analysis (0.5–50 mm; Fig. 2C,D). A single product (UDP‐Xyl) was released and no UDP‐4‐keto‐pentose was detected (Fig. S14), consistent with our previous study [7]. Note, another study reported the release of UDP‐4‐keto‐pentose by hUXS1 when high concentrations of exogenous NAD^+^ were added [13, 23]. More recently, it was demonstrated that the loss of the UDP‐4‐keto‐pentose intermediate can impair hUXS1 activity *in vivo*, leading to Catel–Manzke syndrome, a rare skeletal dysplasia [33].

A detailed analysis of the time course of UDP‐GlcA consumption (Fig. 2B) revealed that, whereas the conversion rates were comparable for the wild‐type and Ile181Asn reactions during the first 10 min (Fig. 2B), after 30 min, the conversion of the wild‐type reaction progressively outpaced that of the Ile181Asn reaction. Ultimately, after 24 h, the conversion reached ~90% in the wild‐type reaction, whereas it leveled out at only ~50% in the Ile181Asn reaction. We also noted the more pronounced formation of protein precipitate in the Ile181Asn reaction (Fig. S15), suggesting that the variant enzyme may not be stable under the conditions used.

### Stability of Ile181Asn‐hUXS1

**Fig. 3:**
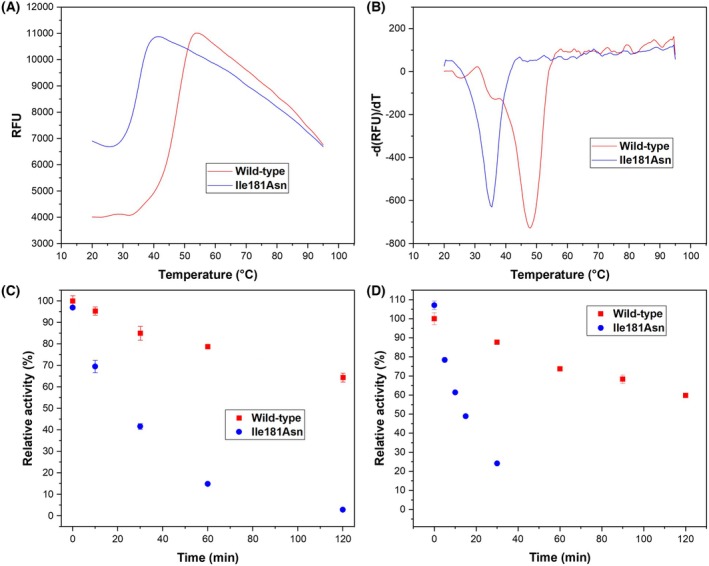
Analysis of the stability of Ile181Asn‐hUXS1. (A) Melt curves and (B) melt peaks obtained from DSF analysis of wild‐type and Ile181Asn‐hUXS1. DSF, differential scanning fluorimetry; RFU, relative fluorescence units. (C, D) Analysis of activity loss at 37 °C during incubation at a protein concentration of 1.0 (C) and 0.10 (D) mg·mL^−1^. Values of the relative activities are average ± SD (*N* = 2), with the initial activity of hUXS1 wild‐type assumed to be 100%. hUXS1, human UDP‐xylose synthase 1.

DSF was used to assess the stability of Ile181Asn‐hUXS1 and wild‐type hUXS1. Results are shown in Fig. 3A,B. With both enzymes, thermal denaturation was indicated by a sharp increase in the fluorescence reporter signal that rose to the maximum value in a continuous course, suggesting cooperative denaturation without the accumulation of intermediates. The apparent melting temperature (*T*_m_) was determined from the temperature derivative plot of the fluorescence signal in dependence on the temperature, as shown in Fig. 3B, where the minimum indicates the *T*_m_. The *T*_m_ of Ile181Asn‐hUXS1 was 35.2 ± 0.3 °C (*N* = 3), while that of wild‐type hUXS1 was considerably higher at 48.2 ± 0.3 °C (*N* = 3). Notably, the *T*_m_ of Ile181Asn‐hUXS1 was even lower than the normal human body temperature (37 °C), suggesting that the variant enzyme may be inherently unstable under physiological conditions.

To further examine differences in stability between Ile181Asn‐hUXS1 and wild‐type enzyme, both enzymes (0.10 and 1.0 mg·mL^−1^) were incubated at 37 °C and the residual activity was measured in dependence of incubation time. Results in Fig. 3C,D show that the activity of Ile181Asn‐hUXS1 declined far more rapidly than the activity of wild‐type hUXS1. The activity loss was described by a single‐exponential decay in both enzymes and the associated rate constants (*k*_in_) were ~10 times higher for Ile181Asn‐hUXS1 (Fig. S7). The inactivation rate was not strongly affected (≤ 1.5‐fold) by protein concentration, suggesting that the activity loss was kinetically first‐order. In both enzymes, the inactivation at 37 °C was accompanied by the formation of protein precipitate. The proportion of total soluble protein lost to solid precipitate (33% for wild‐type hUXS1 after 2 h of incubation; 61% for Ile181Asn‐hUXS1 after 30 min of incubation) closely matched the corresponding loss in enzymatic activity (36% for wild‐type hUXS1 and 57% for Ile181Asn‐hUXS1), indicating a strong correlation between precipitation and activity loss.

To analyze precipitation associated with denaturation of hUXS1, we measured with static light scattering the increase in turbidity due to solids formation during incubation at 37 °C (Fig. S8a). The initial rate of turbidity increase (within 30 min) for Ile181Asn‐hUXS1 was approximately 9–10 times higher than that of wild‐type hUXS1 (Fig. S8b), consistent with their respective inactivation rates (Fig. 3C,D). Compared to the 0.10 mg·mL^−1^ samples, the initial rates of turbidity increase at 1.0 mg·mL^−1^ were approximately 75‐fold and 62‐fold higher for wild‐type hUXS1 and Ile181Asn‐hUXS1, respectively (Fig. S8b). This implies that the formation rate of the precipitated enzyme was approximately 60–80 times faster at a tenfold higher sample concentration, indicating that the precipitation is a process of kinetic order greater than unity and is therefore strongly concentration‐dependent.

### Oligomerization state of Ile181Asn‐hUXS1

**Fig. 4:**
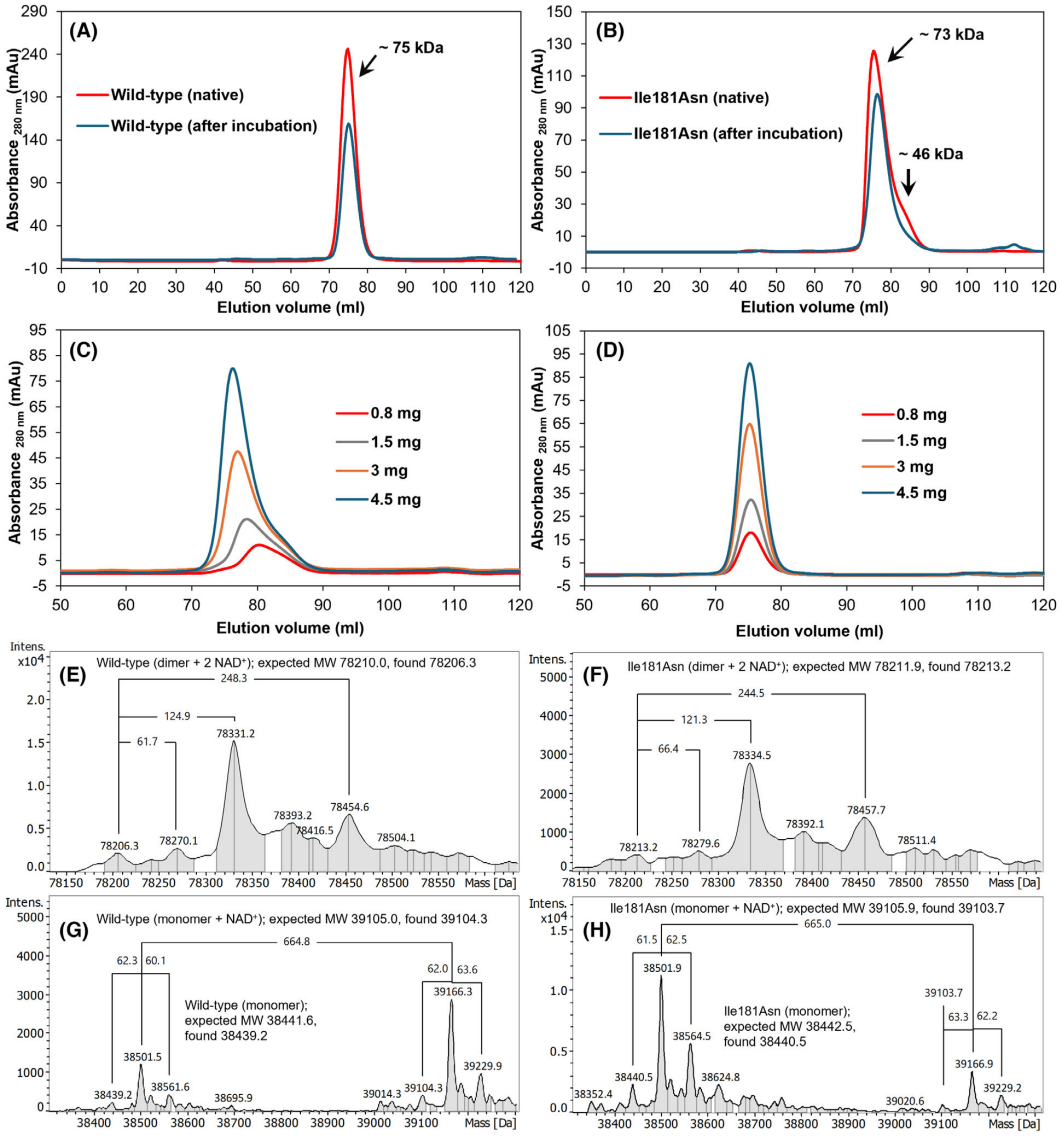
Analysis of the oligomeric state of Ile181Asn‐hUXS1. (A, B) Overlay of SEC (size‐exclusion chromatography) chromatograms for hUXS1 wild‐type (A) and Ile181Asn variant (B) before and after incubation at 37 °C. (C, D) Overlay of SEC chromatograms for Ile181Asn‐hUXS1 (C) and hUXS1 wild‐type (D) with different protein loading (0.8–4.5 mg). (E, F) Deconvoluted spectra of the dimer with NAD^+^ for hUXS1 wild‐type (E) and for Ile181Asn variant (F); (G, H) Deconvoluted spectra of the monomer with and without NAD^+^ for wild‐type hUXS1 (G) and for Ile181Asn variant (H). Note: monomeric and dimeric species may appear as adduct forms, presumed to contain one or more acetate groups (~60 Da), resulting from the use of ammonium acetate in the analysis. hUXS1, human UDP‐xylose synthase 1.

Woods and co‐workers have shown that hUXS1 and the *mow* variant Arg236His‐hUXS1 can populate oligomer states higher than the canonical SDR dimer, dependent on the protein concentration used [23]. Considering the expected destabilization of dimer interface interactions resulting from the substitution of Ile181 by Asn, we were interested in the oligomer state of Ile181Asn‐hUXS1 and applied SEC for its determination. The wild‐type hUXS1 eluted as a single protein peak in SEC, with the elution volume corresponding to a molecular mass (~75 kDa) expected for the dimer (calculated mass: 76884 Da), as shown in Fig. 4A. Higher oligomers were not detected in our analysis. Ile181Asn‐hUXS1 also eluted as an apparent dimer; however, the main peak (71–82 mL) exhibited a shoulder (82–89 mL, ~10% of total protein) at larger elution volumes (Fig. 4B), suggesting the presence of another species of lower apparent mass. The nominal mass of the “shoulder species” (~46 kDa) was close to the expected monomer mass (38 442 Da), possibly indicating a small proportion of monomeric species. As the elution behavior was influenced by the mean of the distribution of monomer and dimer, the minor monomeric fraction appeared larger than its true molecular size. We determined the specific activity of the enzyme eluting in the main peak fraction and in the peak shoulder and found both to be identical (Fig. S10). With decreasing protein loading in the SEC analysis, the elution volume of the main peak for Ile181Asn‐hUXS1 shifted from 76 mL to 80 mL (Fig. 4C), implying that the monomer‐dimer equilibrium of the variant is concentration‐dependent, with lower concentrations favoring dimer dissociation as expected. In contrast, the elution volume of wild‐type hUXS1 remained constant (Fig. 4D) regardless of protein loading, suggesting that the wild‐type dimer is more stable than that of the Ile181Asn variant. Incubation of Ile181Asn‐hUXS1 at 37 °C for 30 min resulted in a decrease in the relative content of the shoulder fraction to below 5% (Fig. 4B). Approximately 60% of the total protein was precipitated during the incubation and removed as a solid before the SEC. The dissociated monomer appears to be more prone to precipitation at 37 °C than the native dimer.

In addition to the SEC analysis, native MS was conducted to examine the oligomerization states of hUXS1 wild‐type and Ile181Asn variant. In contrast to the SEC results, a small proportion of monomeric species (~19%) was detected in the wild‐type enzyme (Table 1), possibly due to partial dissociation upon injection into the MS. The predominance of monomeric species (~84%) in the Ile181Asn‐hUXS1 sample (Table 1) suggests that the dimeric form of the variant is considerably less stable than that of the wild‐type, which is consistent with the reduced overall stability of the variant enzyme. In both hUXS1 wild‐type and Ile181Asn variant, only the holoenzyme form was detected for the dimeric species (Fig. 4E,F), matched with the measured stoichiometric binding of NAD^+^ (1.02 ± 0.04 and 1.07 ± 0.02 mol of NAD^+^ per mol of protein for the wild‐type and Ile181Asn variant, respectively). However, Ile181Asn‐hUXS1 exhibited a relatively higher proportion of monomeric apoenzyme compared to the wild‐type (Fig. 4G,H), suggesting that NAD^+^ binding in the Ile181Asn variant is weaker and may be disrupted during the native MS analysis. This finding is consistent with biochemical data showing that the wild‐type hUXS1 retains approximately 83% of its maximum activity, whereas the Ile181Asn variant exhibits a relatively lower activity (~68%) under assay conditions without externally added NAD^+^ (Fig. S16).

**Table 1:** Results of MS analysis of wild‐type hUXS1 and Ile181Asn variant. hUXS1, human UDP‐xylose synthase 1.

	Mass (Da)^a^	Intensity	Total intensity	Percentage (%)^b^
Wild‐type monomers	38501.5 (+ acetate^c^)	1227	5122	~19
	39166.3 (+ NAD^+^, + acetate)	2904		
	39229.9 (+ NAD^+^, + 2 acetate)	991		
Wild‐type dimers	78331.2 (+ 2 NAD^+^, + 2 acetate)	15 281	22 023	~81
	78454.6 (+ 2 NAD^+^, + 4 acetate)	6742		
Ile181Asn monomers	38501.9 (+ acetate)	11 333	21 942	~84
	38564.5 (+ 2 acetate)	5754		
	39166.9 (+ NAD^+^, + acetate)	3376		
	39229.2 (+ NAD^+^, + 2 acetate)	1479		
Ile181Asn dimers	78334.5 (+ 2 NAD^+^, + 2 acetate)	2810	4207	~16
	78457.7 (+ 2 NAD^+^, + 4 acetate)	1397		

^a^ Predominant monomer and dimer masses in Fig. 4E–H were used to calculate the percentage of each species in wild‐type hUXS1 and Ile181Asn variant.

^b^ The percentages of monomers and dimers in wild‐type and Ile181Asn were calculated as the ratio of the total monomers (or dimers) intensity to the combined intensity of monomers and dimers for each protein. Note that the percentages are approximations based on relative signal intensities.

^c^ The small molecules (~60 Da) observed in the adducts were presumed to be acetate, considering the high acetate concentration of the volatile buffer.

### Molecular analysis of Ile181Asn substitution effects on hUXS1 function and stability

**Fig. 5:**
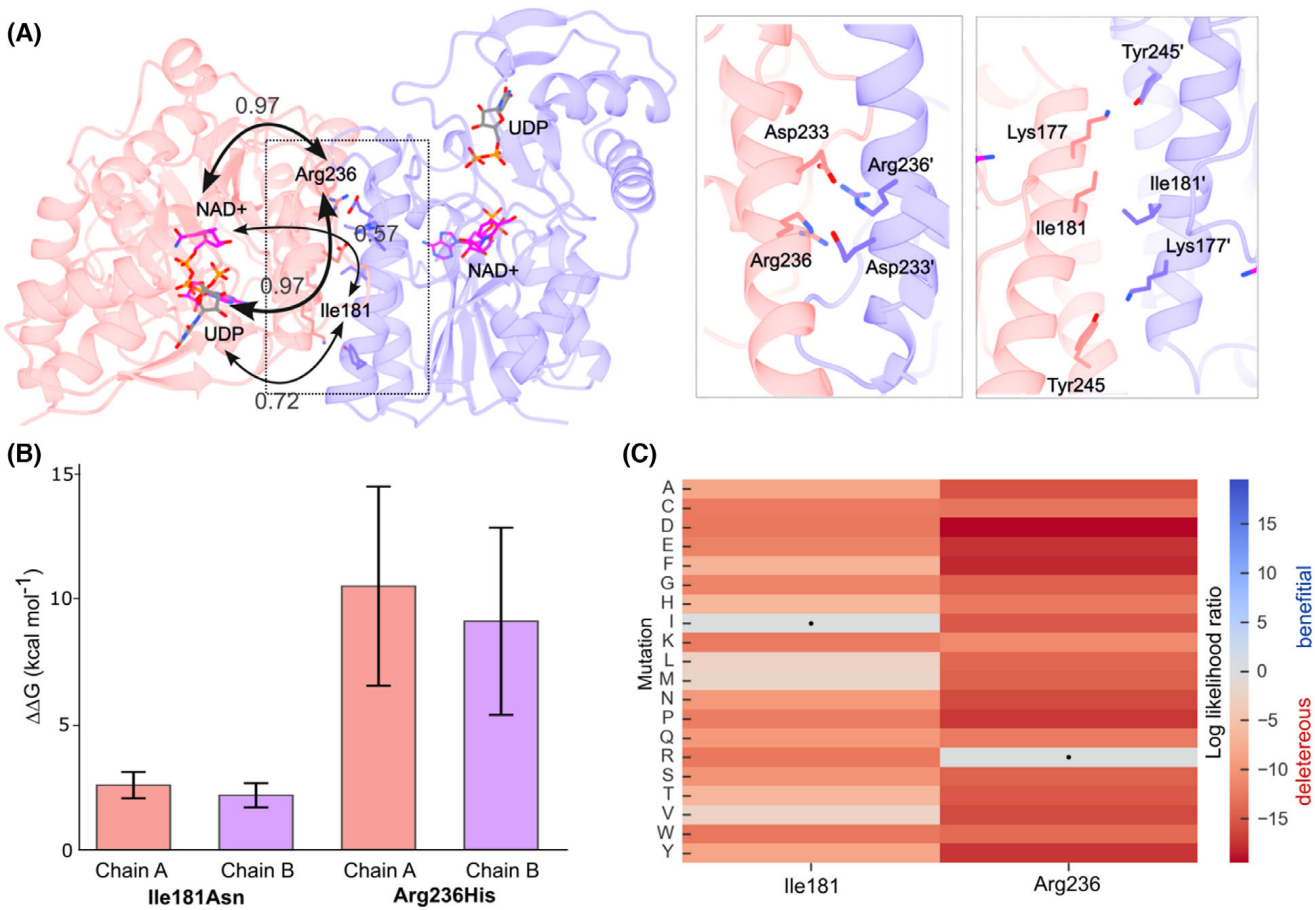
Molecular analysis of the impact of the substitutions Ile181Asn and Arg236His in hUXS1. (A) The hUXS1 dimer and the environment of Ile181 and Arg236 at the dimer interface. The two‐headed arrows indicate the bond‐to‐bond propensity coupling of Ile181 and Arg236 with NAD^+^ and UDP. (B) Change of free energy variation (∆∆G, kcal·mol^−1^) calculated on 500 MD‐simulation snapshots of wild‐type hUXS1 with FoldX 2025. Values of the ∆∆G are means ± SD (*N* = 500 MD simulation snapshots analyzed). (C) Likelihood score for the substitution of positions 181 and 236 by the 20 proteinogenic amino acids obtained with ESM‐1v. hUXS1, human UDP‐xylose synthase 1.

We used bond‐to‐bond propensity analysis [34] to examine structural coupling between the residue at position 181 (Ile, Asn) and the enzyme‐bound ligands NAD^+^ and UDP. The same analysis was performed for the residue at position 236 (Arg, His). The crystallographic structure of hUXS1 (PDB ID: 2B69) was analyzed and results are visualized in Fig. 5A. The bond‐to‐bond coupling score ranges from 0 to 1, where 1 indicates strong coupling between residues in the enzyme. Both residues Ile181 and Arg236 are located at the dimer interface and establish interactions with the other unit. Arg236 has contact with Asp233’ in a bidirectional duplicated interaction. Ile181 interacts with Ile181’ in a hydrophobic environment surrounded by polar residues, including Lys177 and Tyr245. In our analysis, Ile181 and Arg236 differ in their coupling with the ligands at the active site. While position 181 shows a moderate coupling (< 0.73), Arg236 is strongly coupled with both NAD^+^ and UDP (0.97). The bond‐to‐bond propensity analysis supports experimental evidence showing larger disruptive effect on hUXS1 function due to the Arg236His substitution [23] than the Ile181Asn substitution (this work).

Additionally, the dynamics of the hUXS1 dimer bound with NAD^+^ and UDP in each subunit were simulated for 2.5 μs in an aqueous environment. The change in free energy upon site‐specific substitution (ΔΔG) was calculated throughout the MD simulation. Fig. 5B shows the values obtained for the Ile181Asn and Arg236His substitutions in both subunits of the dimer. Both substitutions negatively affect the stability of the enzyme, although to different extents. Based on these data, Ile181Asn may still fold correctly, but small increases in temperature are likely to destabilize the structure. Finally, we predicted the effect of substituting these positions with each of the 20 standard amino acids using ESM‐1v (Fig. 5C). All substitutions are predicted to be deleterious, with a stronger impact observed at position 236.

In conclusion, this study reveals that the Ile181Asn variant of hUXS1 involves impaired protein stability compared to the wild‐type enzyme. The normal hUXS1 catalytic cycle of conversion of UDP‐GlcA into UDP‐Xyl is not affected in the Ile181Asn variant, as evidenced by the lack of release of UDP‐4‐keto‐pentose in the enzymatic reaction *in vitro*, nor are the kinetic parameters (*k*_cat_, *K*_m_) for the reaction altered in the enzyme variant. MD simulations support the experimental data in showing reduced stability of the Ile181Asn variant. The low stability of Ile181Asn‐hUXS1 at body temperature provides a plausible explanation for the proposed functional deficiency of this variant in human physiology [18], leading to the observed disorder of skeletal dysplasia. In other instances of genetic syndrome directly [10, 23] or indirectly [33] linked to hUXS1, however, functional deficiency of hUXS1 originates from impairments of the catalytic cycle. We speculate that Ile181Asn‐hUXS1 might be a target for chaperone therapy [35] wherein a small molecule (chaperone) is used to stabilize and correct misfolding of the unstable enzyme to restore its activity.

## Author contributions

B.N. and T.L. designed the research. T.L. performed the experiments and analyzed the data. P.A.S.‐M. carried out the MD simulations. B.N., T.L., and P.A.S.‐M. wrote the paper.

## Supporting information


**Fig. S1.** Multiple sequence alignment of different hUXS1 transcript variants.
**Fig. S2.** The amino acid sequences of the optimized hUXS1 construct for protein expression.
**Fig. S3.** SDS polyacrylamide gel showing the enzyme isolation.
**Fig. S4.** Time courses of UDP‐Xyl formation for determination of kinetic parameters of hUXS1 wild‐type and Ile181Asn variant at 25 °C.
**Fig. S5.** Determination of kinetic parameters for hUXS1 wild‐type and Ile181Asn variant at 37 °C.
**Fig. S6.** Overlay of HPLC chromatograms comparing the mixture from the Ile181Asn‐hUXS1 reaction with standard samples of UDP‐xylose and UDP‐4‐keto‐xylose.
**Fig. S7.** Natural logarithm (ln) of relative enzymatic activity of the hUXS1 wild‐type and Ile181Asn variant after incubation at 37 °C.
**Fig. S8.** Analysis of precipitation associated with denaturation of hUXS1.
**Fig. S9.** Calibration curve for SEC column prepared with standard mixture.
**Fig. S10.** Time courses of the product (UDP‐Xyl) used to determine the specific activities of proteins from main peak and shoulder peak fractions collected in the SEC (size‐exclusion chromatography) of Ile181Asn‐hUXS1.
**Fig. S11.** Raw spectra of native mass spectrometry analysis for hUXS1 wild‐type and Ile181Asn variant.
**Fig. S12.** Calibration lines for determination of NAD^+^ concentration.
**Fig. S13.** Time courses of the product (UDP‐Xyl) used to determine the specific activities of hUXS1 wild‐type and Ile181Asn variant at 37 °C.
**Fig. S14.** Overlay of HPLC chromatograms comparing the mixtures from the Ile181Asn‐hUXS1 reactions containing different concentration of UDP‐GlcA (2.0–10 mm) with standard sample of UDP‐4‐keto‐xylose.
**Fig. S15.** Images of the hUXS1 wild‐type and Ile181Asn variant reaction mixtures (at 37 °C) taken at 0 min and after 2 h following centrifugation.
**Fig. S16.** Analysis of activities of hUXS1 wild‐type and Ile181Asn variant with different concentration of NAD^+^.

## Data Availability

The MD simulation data are available at https://doi.org/10.5281/zenodo.17511760. The experimental data are reported in the paper.
