# Supplementary material for: Structural instability impairs function of the UDP‐xylose synthase 1 Ile181Asn variant associated with short‐stature genetic syndrome in humans
Source: FEBS Lett. 2026 Jan 13;600(14):2063–76. doi: 10.1002/1873-3468.70277 (PMC13404148; doi:10.1002/1873-3468.70277)
Supplement: Supplementary file 1 — Fig. S1. Multiple sequence alignment of different hUXS1 transcript variants. Fig. S2. The amino acid sequences of the optimized hUXS1 construct for protein expression. Fig. S3. SDS polyacrylamide gel showing the enzyme isolation. Fig. S4. Time courses of UDP‐Xyl formation for determination of kinetic parameters of hUXS1 wild‐type and Ile181Asn variant at 25 °C. Fig. S5. Determination of kinetic parameters for hUXS1 wild‐type and Ile181Asn variant at 37 °C. Fig. S6. Overlay of HPLC chromatograms comparing the mixture from the Ile181Asn‐hUXS1 reaction with standard samples of UDP‐xylose and UDP‐4‐keto‐xylose. Fig. S7. Natural logarithm (ln) of relative enzymatic activity of the hUXS1 wild‐type and Ile181Asn variant after incubation at 37 °C. Fig. S8. Analysis of precipitation associated with denaturation of hUXS1. Fig. S9. Calibration curve for SEC column prepared with standard mixture. Fig. S10. Time courses of the product (UDP‐Xyl) used to determine the specific activities of proteins from main peak and shoulder peak fractions collected in the SEC (size‐exclusion chromatography) of Ile181Asn‐hUXS1. Fig. S11. Raw spectra of native mass spectrometry analysis for hUXS1 wild‐type and Ile181Asn variant. Fig. S12. Calibration lines for determination of NAD+ concentration. Fig. S13. Time courses of the product (UDP‐Xyl) used to determine the specific activities of hUXS1 wild‐type and Ile181Asn variant at 37 °C. Fig. S14. Overlay of HPLC chromatograms comparing the mixtures from the Ile181Asn‐hUXS1 reactions containing different concentration of UDP‐GlcA (2.0–10 mm) with standard sample of UDP‐4‐keto‐xylose. Fig. S15. Images of the hUXS1 wild‐type and Ile181Asn variant reaction mixtures (at 37 °C) taken at 0 min and after 2 h following centrifugation. Fig. S16. Analysis of activities of hUXS1 wild‐type and Ile181Asn variant with different concentration of NAD+. [file FEB2-600-2063-s001.pdf]

Supporting Information

Structural instability impairs function of the UDP-xylose synthase 1 Ile181Asn variant associated with short-stature genetic syndrome in humans

Tuo Li[1], Pedro A. Sánchez-Murcia[2,3] and Bernd Nidetzky[1,4,*]

[1]Institute of Biotechnology and Biochemical Engineering, Graz University of Technology, NAWI Graz, Petersgasse 12, Graz, Austria

[2]Division of Medicinal Chemistry, Otto-Loewi Research Center, Medical University of Graz, Neue Stiftingtalstraße 6, Graz, Austria

[3]BioTechMed-Graz, Mozartgasse 12, Graz, Austria

[4]Austrian Centre of Industrial Biotechnology (acib), Krenngasse 37, Graz, Austria

* Corresponding author (B.N.): bernd.nidetzky@tugraz.at

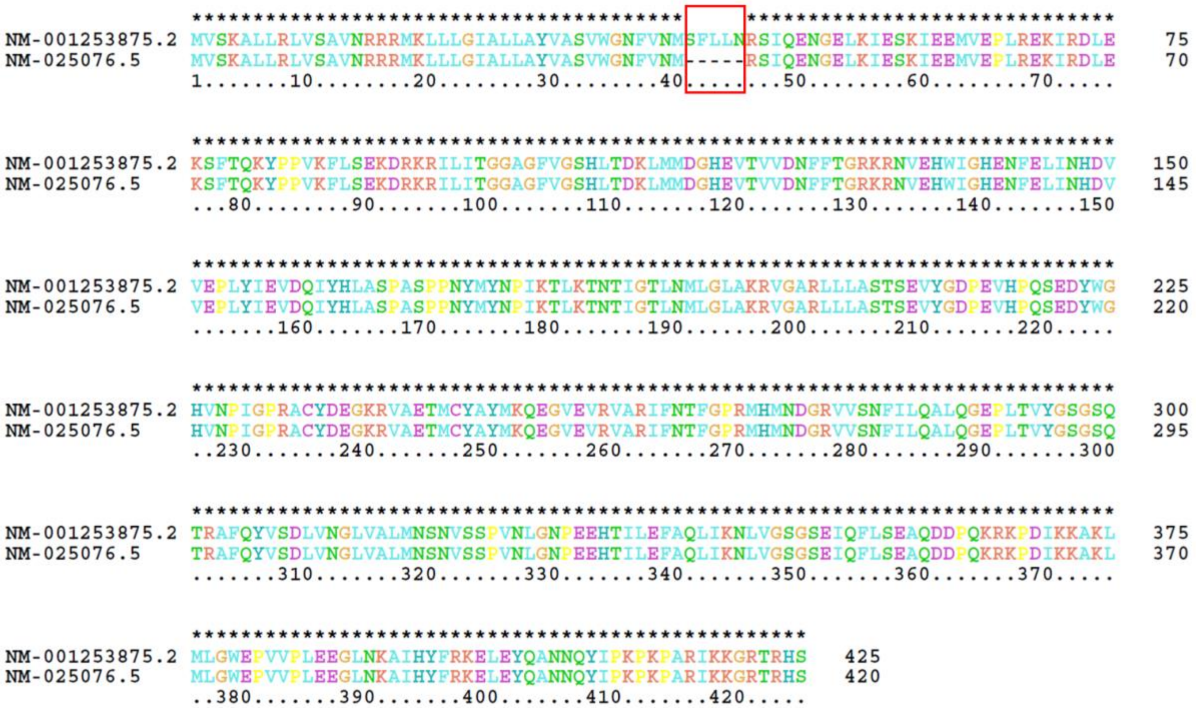

Figure S1. Multiple sequence alignment of different hUXS1 (human UDP-xylose synthase 1) transcript variants. Splice variant (NM_001253875.2, NCBI Reference Sequence: NP_001240804.1) utilizes an alternative in-frame splice site in the 5′-coding region compared to native transcript (NM_025076.5, NCBI Reference Sequence: NP_079352.2), resulting in the insertion of five additional amino acids (SFLLN, marked in red box) after residue 40. Multiple sequence alignment was executed using ClustalX software.

Protein sequence of truncated hUXS1 with N-terminal 6×His-tag and a tobacco etch virus (TEV) protease cleavage site

MHHHHHHSSGVDLGTENLYFQSME[85]KDRKRILITGGAGFVGSHLTDKLMMDGHEV
TVVDNFFTGRKRNVEHWIGHENFELINHDVVEPLYIEVDQIYHLASPASPPNYMYNPI
KTLKTNTI[181]GTLNMLGLAKRVGARLLLASTSEVYGDPEVHPQSEDYWGHVNPIGPR
ACYDEGKRVAETMCYAYMKQEGVEVRVARIFNTFGPRMHMNDGRVVSNFILQALQ
GEPLTVYGSGSQTRAFQYVSDLVNGLVALMNSNVSSPVNLGNPEEHTILEFAQLIKN
LVGSGSEIQFLSEAQDDPQKRKPDIKKAKLMLGWEPVVPLEEGLNKAIHYFRKELEY
QANNQ[402]

Figure S2. The amino acid sequences of the optimized hUXS1 (human UDP-xylose synthase 1) construct for protein expression. The N-terminal 6×His tag is highlighted in yellow, the truncated hUXS1 protein sequence is shown in grey (aa 85 – 402), and Ile181 is marked in red.

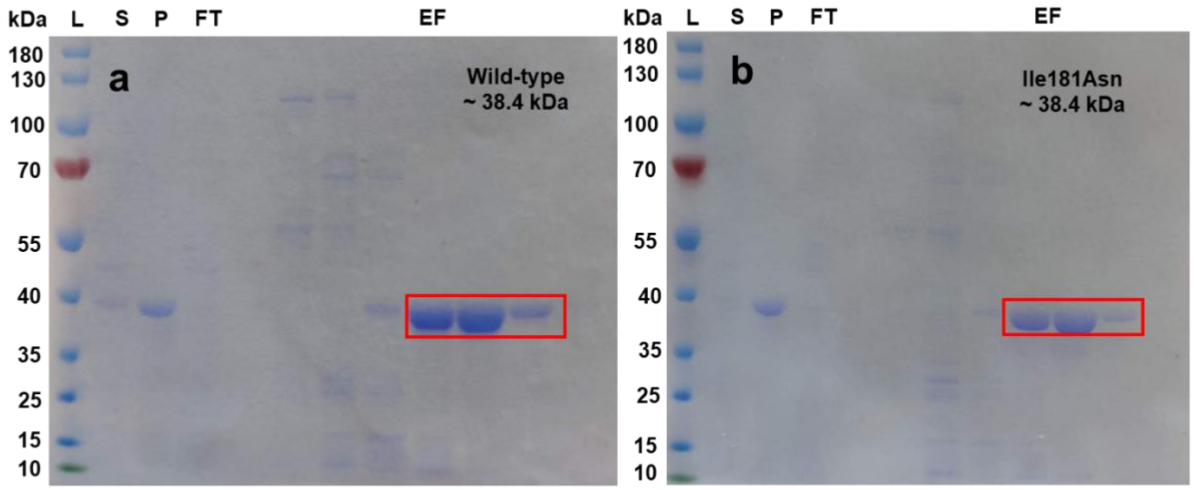

Figure S3. SDS polyacrylamide gel showing the enzyme isolation. Wild-type hUXS1 (**a**) and Ile181Asn-hUXS1 (**b**). L, molecular mass marker; S, supernatant; P, pellet; FT, flow through (unbound proteins); EF, elution fractions. A substantial portion of expressed protein was present in the insoluble pellet (see the P lane). hUXS1, human UDP-xylose synthase 1.

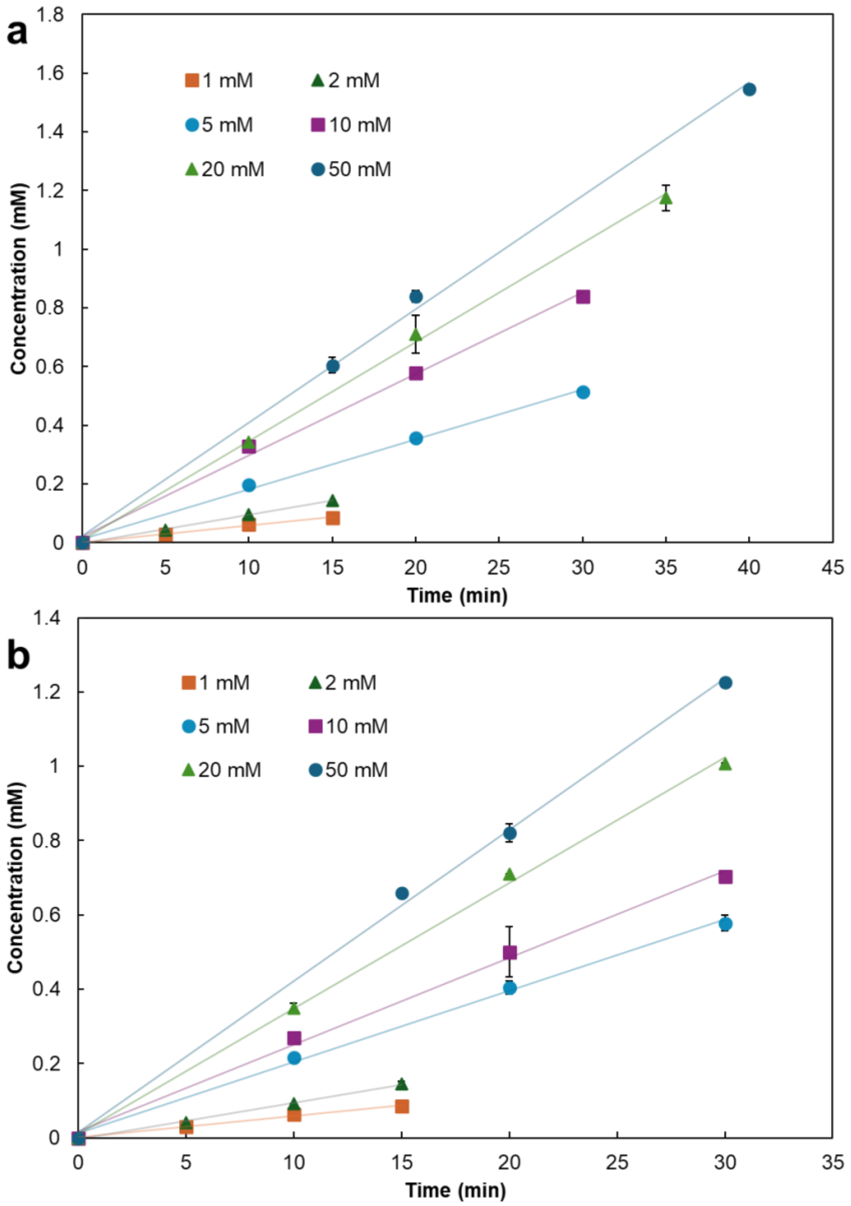

Figure S4. Time courses of UDP-Xyl (UDP-xylose) formation in wild-type hUXS1 (**a**) and Ile181Asn variant (**b**) reactions, carried out using varying concentrations of UDP-GlcA (UDP-glucuronic acid) to determine kinetic parameters. Assays were conducted at 25 °C and contained 0.20 mg/ml enzyme, 1.0 – 50 mM UDP-GlcA, and 0.50 mM NAD^+. Values of the concentration are average ± SD ($N = 2$). hUXS1, human UDP-xylose synthase 1.

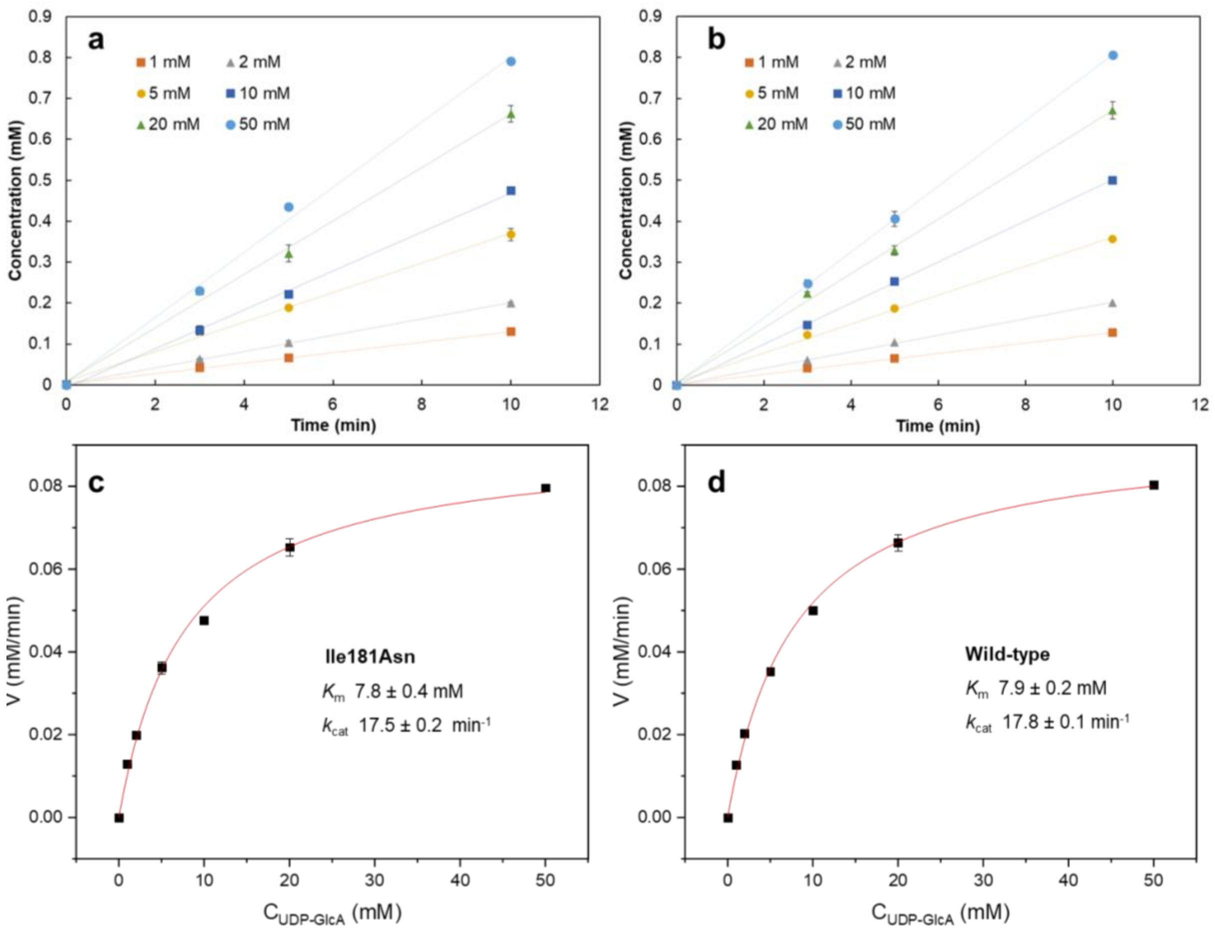

Figure S5. Determination of kinetic parameters for hUXS1 wild-type and Ile181Asn variant at 37 °C. (**a, b**) Time courses of UDP-Xyl (UDP-xylose) formation in Ile181Asn-hUXS1 (**a**) and hUXS1 wild-type (**b**) reactions with varying concentrations of UDP-GlcA (UDP-glucuronic acid). Values of the concentration are average ± SD (N = 2). (**c**, **d**) Michaelis-Menten plot for Ile181Asn-hUXS1 (**c**) and hUXS1 wild-type (**d**). Assays contained 0.20 mg/ml enzymes, 1.0 – 50 mM UDP-GlcA, 0.50 mM NAD^+, and were performed at 37 °C. hUXS1, human UDP-xylose synthase 1.

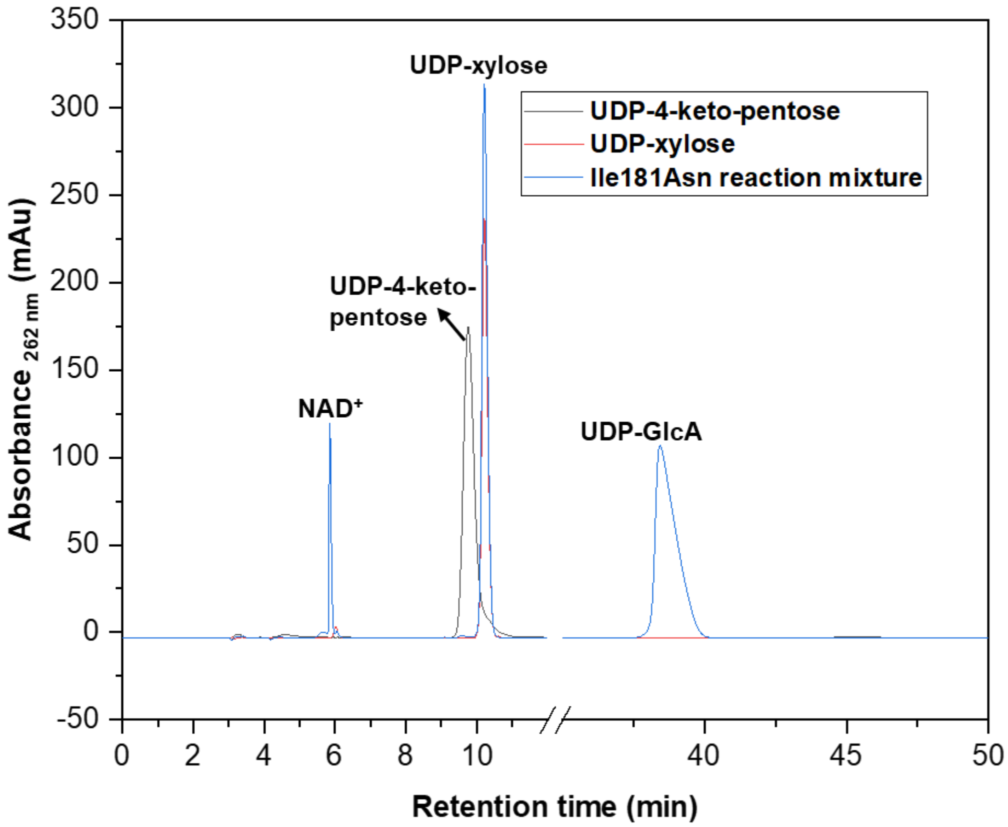

Figure S6. Overlay of HPLC chromatograms comparing the mixture from the Ile181Asn-hUXS1 reaction with standard samples of UDP-xylose and UDP-4-keto-pentose. UDP-GlcA, UDP-glucuronic acid; hUXS1, human UDP-xylose synthase 1.

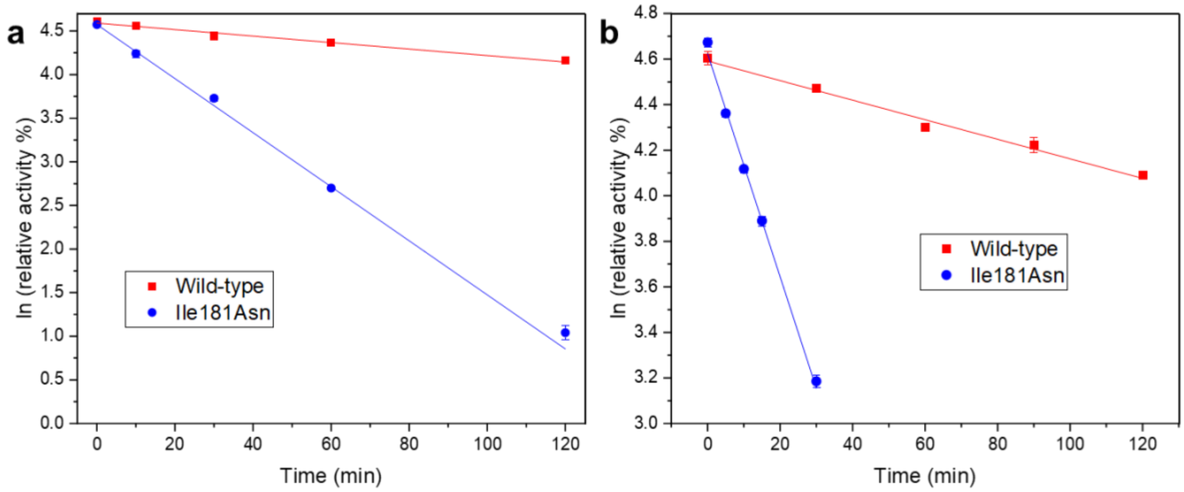

Figure S7. Natural logarithm (ln) of relative enzymatic activity of the hUXS1 wild-type and Ile181Asn variant after incubation at 37 °C, using protein concentrations of 1.0 mg/ml (**a**) and 0.10 mg/ml (**b**). The associated rate constants (k_{in}) were determined as $3.7 \times 10^{-3} \pm 1.9 \times 10^{-4}$ min^{-1} (1.0 mg/ml), $4.3 \times 10^{-3} \pm 3.3 \times 10^{-4}$ min^{-1} (0.10 mg/ml) for wild-type hUXS1, and $3.1 \times 10^{-2} \pm 5.2 \times 10^{-4}$ min^{-1} (1.0 mg/ml), $4.9 \times 10^{-2} \pm 1.9 \times 10^{-3}$ min^{-1} (0.10 mg/ml) for Ile181Asn-hUXS1. hUXS1, human UDP-xylose synthase 1.

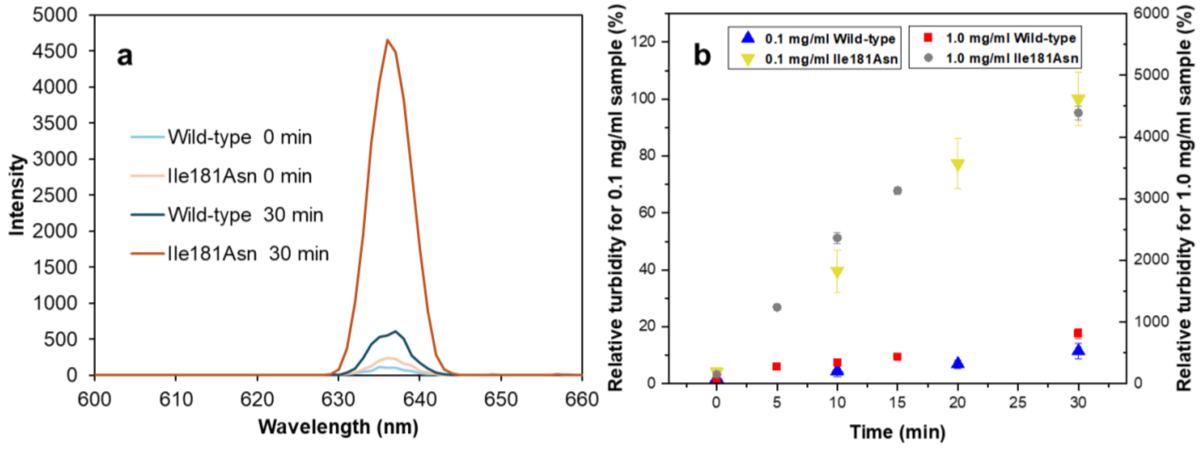

Figure S8. Analysis of precipitation associated with denaturation of hUXS1. (**a**) Overlay of excitation spectra of samples of hUXS1 wild-type and Ile181Asn variant (0.10 mg/ml) before and after incubation at 37 °C. (**b**) Time courses for the relative turbidity of solutions of hUXS1 wild-type and Ile181Asn variant. Different Y-axis scales were used for 0.10 mg/ml and 1.0 mg/ml samples to clearly show the results. Values of the relative turbidity are average ± SD (*N* = 3), with the static light scattering of Ile181Asn-hUXS1 (0.10 mg/ml) at 30 min assumed to be 100% turbidity. hUXS1, human UDP-xylose synthase 1.

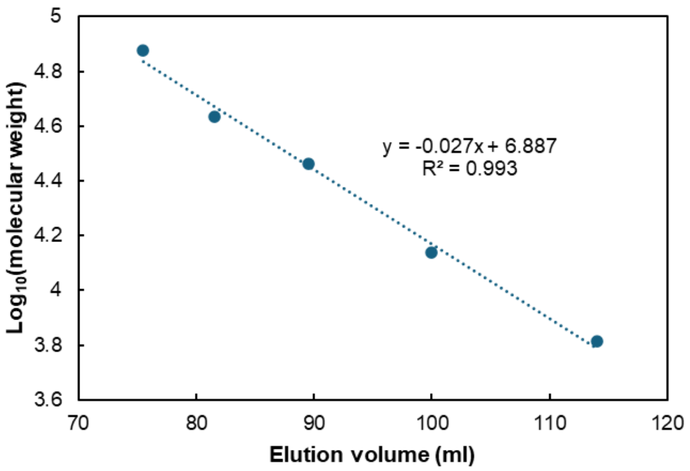

Figure S9. Calibration curve for SEC (size-exclusion chromatography) column prepared with standard mixture. The logarithm of the molecular masses of conalbumin (75000 Da), ovalbumin (43000 Da), carbonic anhydrase (29000 Da), ribonuclease A (13700 Da) and aprotinin (6500 Da) is plotted against their elution volumes.

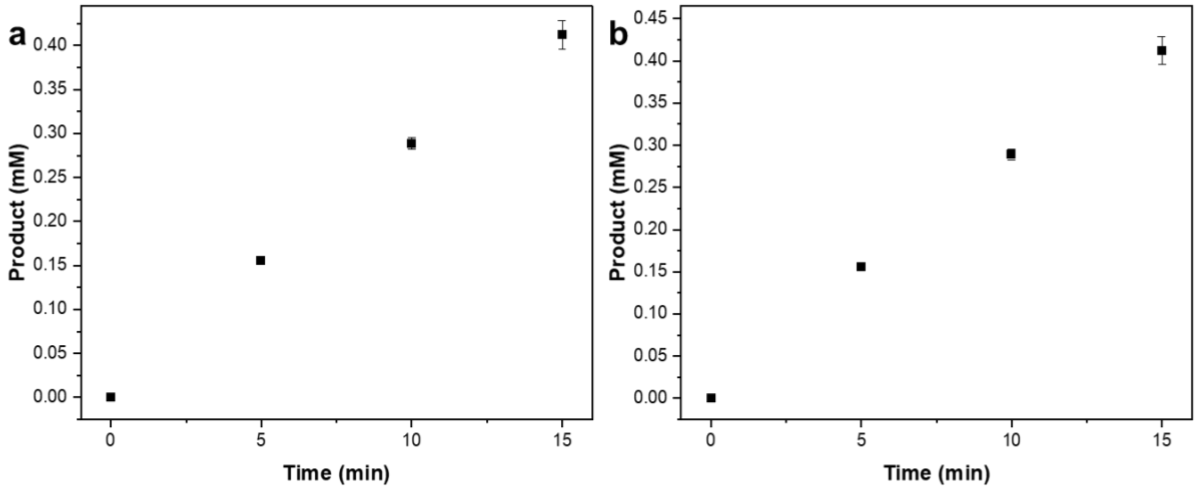

Figure S10. Time courses of the product (UDP-xylose) used to determine the specific activities of proteins from main peak (**a**) and shoulder peak (**b**) fractions collected in the SEC (size-exclusion chromatography) of Ile181Asn-hUXS1. Reactions (100 µl) contained enzymes (0.30 mg/ml), 5.0 mM UDP-GlcA, 0.50 mM NAD^+, and were performed at 25 °C. Values of the product concentration are mean values ± SD ($N = 2$). The activities with UDP-GlcA (92 ± 2.1 mU/mg for **a**, 94 ± 1.4 mU/mg for **b**) were calculated based on initial product (UDP-xylose) formation (0 – 15 min). One unit (U) of enzymatic activity is defined as the amount of enzyme that produces 1 µmol of UDP-xylose per minute.

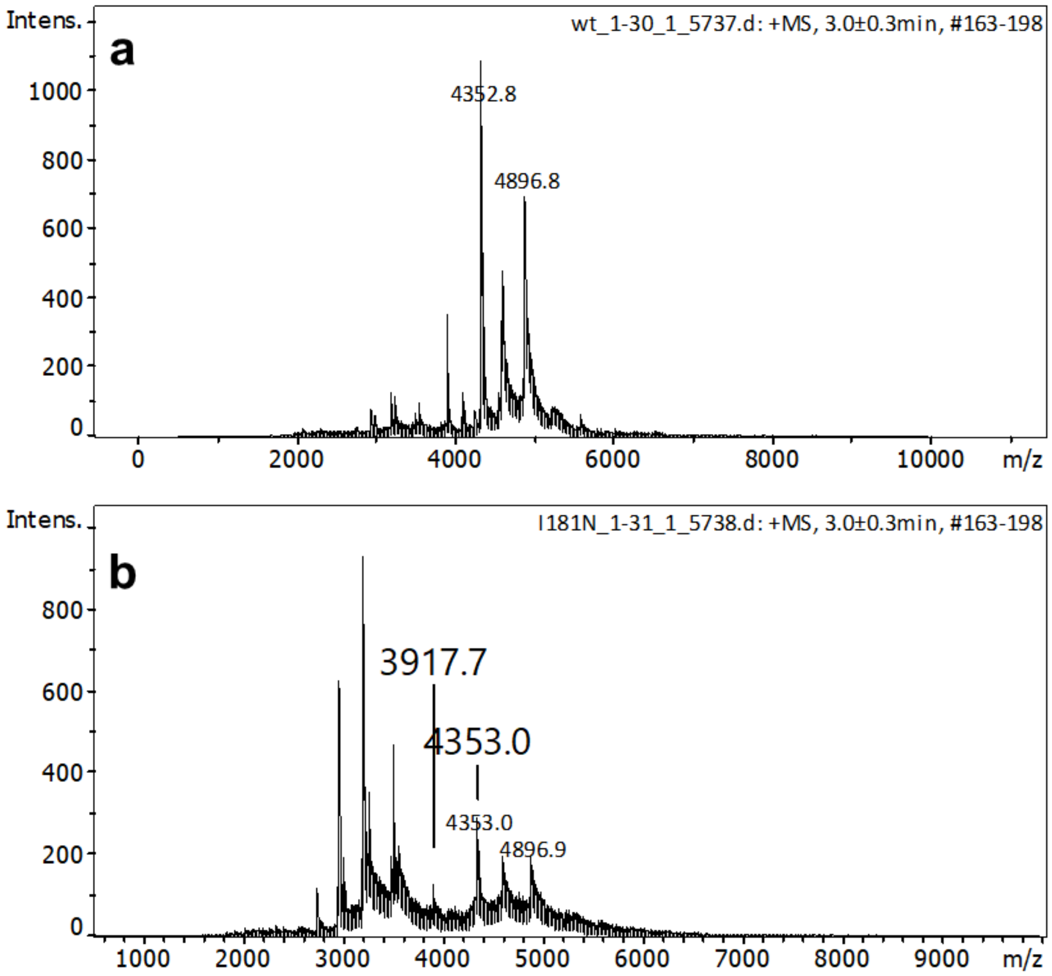

Figure S11. Raw spectra of native mass spectrometry analysis for hUXS1 wild-type (**a**) and Ile181Asn variant (**b**). hUXS1, human UDP-xylose synthase 1.

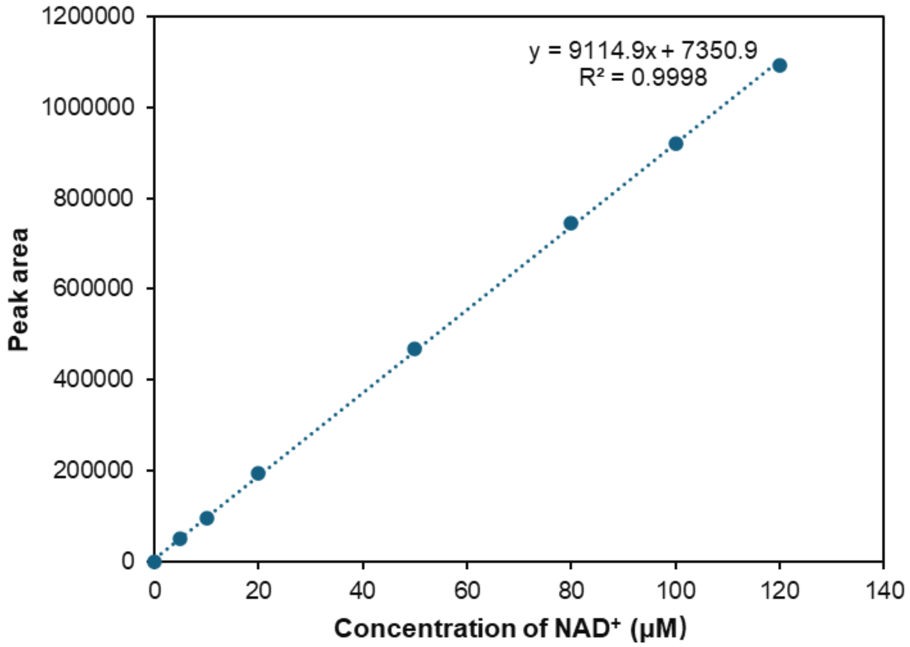

Figure S12. Calibration lines for determination of NAD^+ concentration. The relationship between the concentration of NAD^+ and corresponding HPLC integrated peak areas (at 262 nm) for the measurement of the concentration of NAD^+ in enzymes.

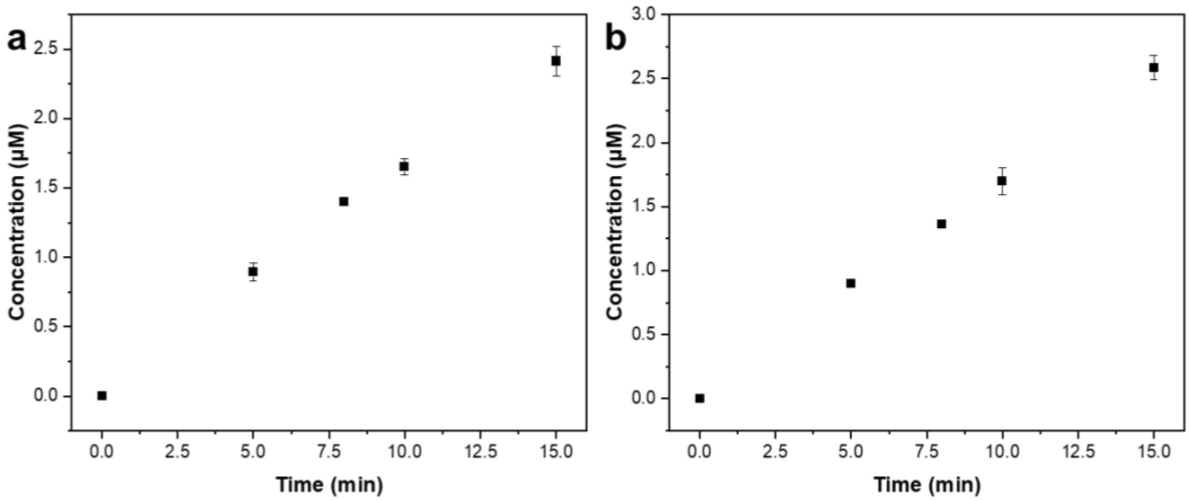

Figure S13. Time courses of the product (UDP-xylose) used to determine the specific activities of hUXS1 wild-type (**a**) and Ile181Asn variant (**b**). The activities with UDP-GlcA (0.81 ± 0.03 mU/mg for hUXS1 wild-type, 0.85 ± 0.04 mU/mg for Ile181Asn-hUXS1) were calculated based on initial product (UDP-xylose) formation (0 – 15 min). Reactions (50 µl) contained enzymes (0.20 mg/ml), 20 µM UDP-GlcA (UDP-glucuronic acid), 2.5 mM NAD^+ in phosphate-buffered saline (PBS) buffer, and were performed at 37 °C. Values of the concentration are average ± SD ($N = 2$). hUXS1, human UDP-xylose synthase 1.

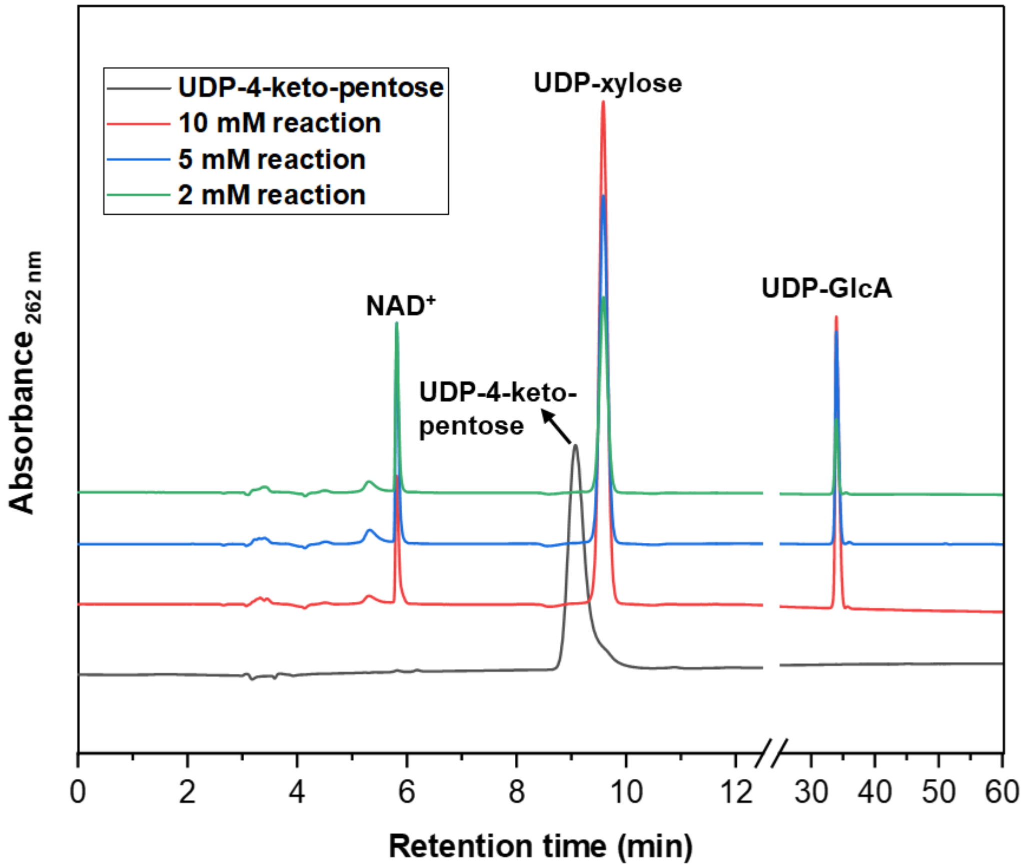

Figure S14. Overlay of HPLC chromatograms comparing the mixtures from the Ile181Asn-hUXS1 reactions containing different concentration of UDP-GlcA (UDP-glucuronic acid, 2.0 – 10 mM) with standard sample of UDP-4-keto-pentose.

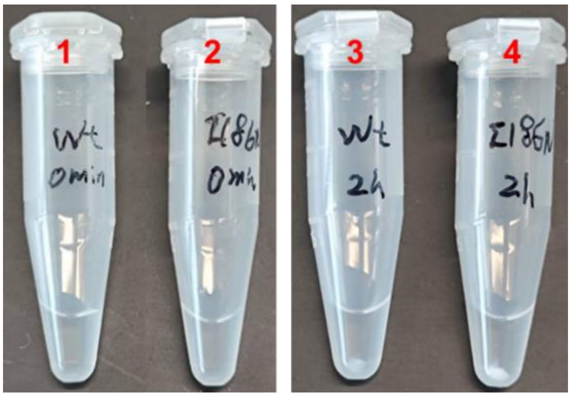

Figure S15. Images of the hUXS1 wild-type (**1**, **3**) and Ile181Asn variant (**2**, **4**) reaction mixtures (at 37 °C) taken at 0 min (**1**, **2**) and after 2 h (**3**, **4**), following centrifugation at 4 °C, 15,000 rpm for 10 minutes. hUXS1, human UDP-xylose synthase 1.

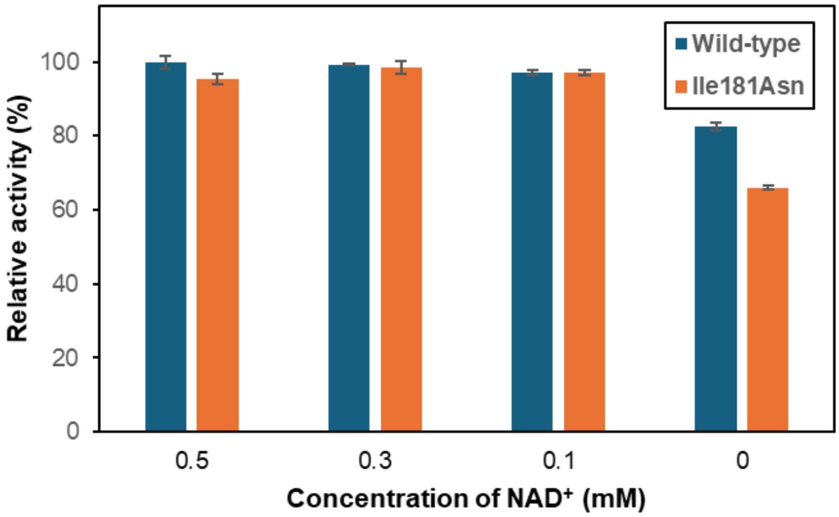

Figure S16. Analysis of activities of hUXS1 wild-type and Ile181Asn variant with different concentration of NAD^+. Assays contained 0.39 mg/ml (10 μM) enzymes, 2.0 mM UDP-GlcA (UDP-glucuronic acid), 0 – 0.50 mM NAD^+, and were performed at 25 °C. Values of the relative activities are average ± SD ($N = 2$), with the activity of hUXS1 wild-type with 0.50 mM NAD^+ assumed to be 100%. hUXS1, human UDP-xylose synthase 1.
